# Physical Activity and Cognitive Aspects of Self-Regulation in Preschool-Aged Children: A Systematic Review

**DOI:** 10.3390/ijerph17186576

**Published:** 2020-09-09

**Authors:** Aaron P. Wood, Vincenzo G. Nocera, Tyler J. Kybartas, Dawn P. Coe

**Affiliations:** Department of Kinesiology, Recreation, and Sport Studies, University of Tennessee, Knoxville, TN 37996, USA; vnocera@vols.utk.edu (V.G.N.); tjkybar@ilstu.edu (T.J.K.); dcoe@utk.edu (D.P.C.)

**Keywords:** preschool children, young children, attention, working memory, inhibition, physical activity

## Abstract

Previous research showed a positive relationship between physical activity and self-regulation in older children and adolescents, but few publications focused on young children. Therefore, the purpose of this review was to examine the impact of physical activity (PA) on the cognitive aspects of self-regulation (inhibition, attention, and working memory, in preschool-aged (3–6 years old) children. The following databases were searched for articles: PubMed, SPORTDiscus, PsycINFO, and ERIC. References of the reviewed papers were screened for the identification of additional articles to be included in the review. Randomized control trials were reviewed to assess the impact of PA interventions on the cognitive aspects of self-regulation. There were six articles included in this review. Each study assessed at least one cognitive aspect of self-regulation [attention (*n* = 4), working memory (*n* = 2), and inhibition (*n* = 5)]. Attention was the only aspect that consistently showed improvement as a result of the PA interventions (4 of 4 studies). Only one study showed a positive impact on working memory and 60% of studies (3 of 5 studies) reported a positive relationship between PA and inhibition. Overall, the findings from this review showed that a consistent relationship only existed between attention and PA. No clear relationships were evident between working memory and inhibition and PA.

## 1. Introduction

The Physical Activity Guidelines for Americans recommend that preschool-aged children (3–6 years old) engage in physical activity (PA) throughout the day, with an emphasis on active play, which is developmentally appropriate and includes a variety of activity intensities [1]. PA in early childhood promotes general growth and development and there is an increasing focus on the relationship between PA and the benefits on the brain and cognitive function. PA was shown to improve cognitive function [2], executive function [3], working memory [4], the structure and function of the brain [5], and reduce depression and anxiety [6]. The development of the brain depends on the health of the body and is especially important in young children to foster mental and intellectual potential [7]. PA was shown to be positively correlated with school readiness in preschoolers and academic achievement in school-aged youth. Children’s behaviors in the classroom and self-regulatory skills, including working memory and attention, are also strong predictors of academic achievement [8].

### 1.1. Self-Regulation

Exploring these mental processes involves the exploration of executive function and self-regulation. Executive function is classified as the collective of cognitive abilities that coordinate and control a set of cognitive processes that is needed to respond to the cues of environmental demands [9]. Self-regulation, a term that falls under the umbrella of executive function, is classified as a higher order cognitive process—referring to the capacity to direct and control one’s actions and attention [8]. Self-regulation can be applied to a number of different contexts, including behaviors, emotions, and cognition. The *Handbook of Self-Regulation* states that it is not only difficult to define self-regulation but is also challenging to operationalize and assess the construct. The varying contexts of self-regulation and the lack of consistency in definitions and assessments in research create difficulty in making direct comparisons. Therefore, researchers need to be deliberate in the choice of context and assessments of self-regulation [10]. This review specifically focuses on the cognitive aspect of self-regulation. Each of the three cognitive aspects of self-regulation (attention, working memory, and inhibition) play a pivotal role in the development and assessment of self-regulation. Attention is widely understood to be an individual’s ability to maintain focus on a particular task. In addition to the ability to maintain focus, another aspect of defining attention, is the ability to shift that focus at will. Diamond uses the term “cognitive flexibility,” defined as “the ability to flexibly switch perspectives, focus of attention, or response mapping.” This term can be used interchangeably in the context that term attention is defined in the current review [11]. Working memory is defined as a system for temporarily storing and managing the information required to carry out complex cognitive tasks, such as learning, reasoning, and comprehension. Lastly, inhibition, which is the most commonly assessed aspect of self-regulation, is defined as the ability to exercise restraint on the direct expression of an instinct or maintain focus on a relevant or intended cue while ignoring an irrelevant or unintended cue. Self-regulation was shown to be linked to both short-term and long-term academic success [12]. Assessments of self-regulation appear to be strong predictors of performance on standardized tests in school-aged youth [13]. Self-regulatory skills begin to emerge during early childhood and continue to develop as a child ages. Therefore, the development of self-regulatory skills and the assessment of self-regulation, might be a proxy of future academic achievement in school-aged youth.

### 1.2. Assessment of Self-Regulation 

In the literature, studies that included all three cognitive aspects of self-regulation have typically done so through some combination of tests that measured each aspect individually and then combined those results to determine the level of the child’s self-regulation. Others have measured a combination of either only working memory and inhibition or included only one of the cognitive aspects of self-regulation. To the authors knowledge, studies that included all cognitive aspects of self-regulation in a single assessment are limited. In the preschool-aged population, many of the self-regulatory assessments that are utilized to assess one or more cognitive aspects of self-regulation were validated in older youth and adults and were adapted to a format that could be completed by preschool-aged children. Due to the use of a variety of assessment tools to measure self-regulation and the three individual cognitive aspects, the lack of measurement consistency might impact the ability to determine the associations and relationships among PA and the cognitive aspects of self-regulation.

### 1.3. Physical Activity and Self-Regulation 

Carson et al. published a review paper summarizing the impact of PA on cognitive development in young children [14]. Additionally, other reviews were published that focused on older children and adolescents, and the relationship between PA, cognitive function, and development [15,16]. Although these reviews were not specific to self-regulation or its cognitive aspects, they each included at least one of the cognitive aspects of self-regulation in the reviews. Overall, these reviews indicated that there is a positive relationship between PA, cognitive function, and development in youth. To date, no review paper specifically looking at the relationship between PA and self-regulation in preschool-aged children is published. There is a small but growing body of evidence, though weak, that examined the possible associations/relationships of these variables [9]. Therefore, there is a need for review to specifically explore the cognitive aspects of self-regulation and the methods of testing, to add to the growing body of research and understanding in this area. Therefore, the objective of this study was to examine the impact that PA has on the cognitive aspects of self-regulation in a preschool-aged (3–6 years old) population. Randomized control trials were reviewed to assess the impact of PA interventions on the cognitive aspects of self-regulation.

## 2. Methods

To examine the relationship between self-regulation and PA in preschool-aged children, a systematic review of published literature was conducted from October 2018 through January 2020. The Preferred Reporting Items for Systematic reviews and Meta-Analysis (PRISMA) statement was used to guide the systematic search and report results [17].

### 2.1. Inclusion/Exclusion Criteria/Analysis 

There were four (4) key criteria for studies to be included in the review: (1) The published study was written in English; (2) the participants were young children in the preschool age range (3–6 years old); (3) at least one aspect of self-regulation was measured using child input, meaning the child completed the assessment; and (4) there was a PA intervention. The initial database search yielded 1147 articles. After the removal of the duplicate titles, the abstracts and papers were screened in full to see if they met the inclusion criteria. There was a total of six articles that were included in this review (See Table 1).

### 2.2. Population 

Studies had to include preschool children, aged 3–6 years old, who were apparently healthy and without any physical or cognitive limitations or disabilities that would restrict PA or the ability to complete the self-regulation assessment. For the studies included in the review, the type of preschool (private versus public) was evenly divided, the socioeconomic status of the preschool communities ranged from low income to middle-to-high income, and the geographical environment (urban versus rural) and location (international sites) varied. It was not noted in any of the studies that formal physical education was offered at these preschools.

### 2.3. Exposure 

The exposure variable was PA. PA was assessed using objective measures, such as accelerometry, or was quantified in an intervention. Subjective measures of PA (e.g., parent proxy, questionnaire) were not included. Additionally, measures of physical fitness were not considered to be a proxy for PA levels.

### 2.4. Outcome

With regards to self-regulation, either all three cognitive aspects were measured simultaneously in one test or any one cognitive aspect (attention, working memory, or inhibitory control) was considered to be an outcome variable of interest. At least one aspect of self-regulation was measured using child input, meaning the child completed the assessment, to be included in the review. Only product-orientated assessment outcomes were included. Parent/caregiver proxy reports of self-regulation were not eligible to be included.

### 2.5. Search Strategy 

PubMed, SPORTDiscus, PsycINFO, and ERIC databases were used to collect peer-reviewed articles on self-regulation and PA in preschool-aged children. There were no restrictions based on the date when the articles were published, due to the limited published literature in this area. The search terms were—“physical activity” and “preschool,” where used in combination with the terms “inhibition,” “working memory,” “attention,” “self-regulation,” along with the combination of “inhibition + executive function,” “working memory + executive function,” “attention + executive function,” and “self-regulation + executive function.” Using these search combinations, 32 individual searches were conducted (8 per search engine). References of the reviewed papers were screened for the identification of additional articles to be included in the review.

### 2.6. Data Extraction 

Data were extracted from each article that was included in the study. The primary author (APW) extracted the data from the articles, and then these data were verified by two independent reviewers/secondary authors (TJK, DPC). Data were entered into an Excel spreadsheet—author/publication year, sample size and participant characteristics, information related to the cognitive aspects of self-regulation assessed (which cognitive aspects and the assessment tool), information related to the PA exposure (intervention), main findings, risk of bias, and overall impact.

### 2.7. Data Synthesis 

A narrative analysis was performed using the studies included in this review. This type of analysis was conducted due to the variation in the exposure and outcome measures used in the studies. The studies were grouped by the cognitive aspect(s) of self-regulation that were assessed—(1) attention; (2) working memory; and (3) inhibition. Within each of these areas, the PA exposure (objective assessment or quantified in an intervention) was identified in the context of each study. For each study included in the narrative analysis, the authors deemed the study to have a “positive impact,” “negative impact,” or “no impact.” These classifications reflect the impact of PA on each component of self-regulation assessed during each individual study.

### 2.8. Study Quality Assessment: Risk of Bias 

After selecting the studies to be included in the review, the risk of bias was assessed for each individual study. The risk of bias assessment was completed using the revised Cochrane risk-of-bias tool for randomized trials [18]. This tool was used to classify each study as low, high, or unclear risk of bias, based on the following criteria—random sequence generation, allocation concealment, blinding of participants and personnel, blinding of outcome assessment, incomplete outcome data, selecting reporting, and other bias. Risk of bias for each study was assessed by one reviewer and verified by another reviewer. Overall risk of bias for each of the studies was also determined. This information is included in Table 2.

## 3. Results

**Figure 1:**
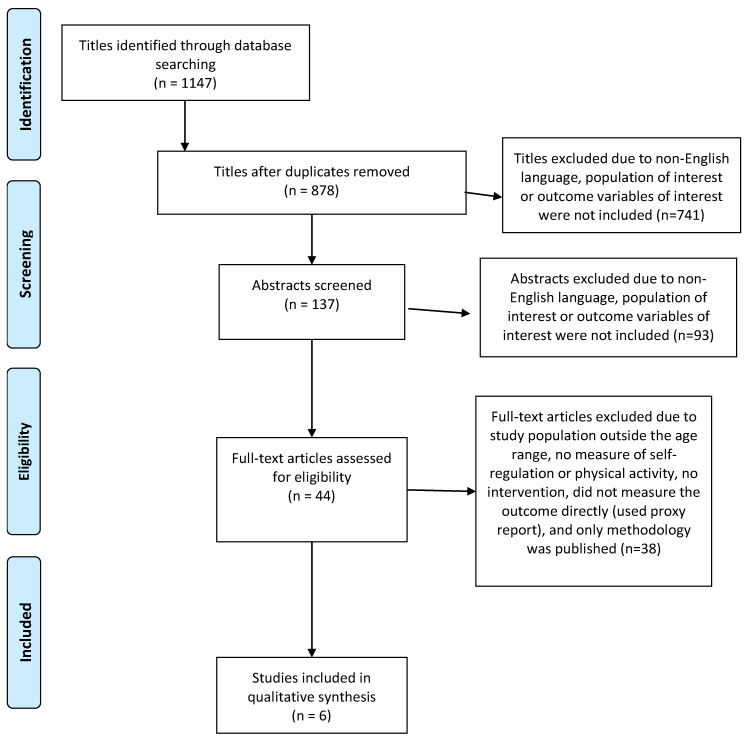
Study identification flow diagram.

The initial search resulted in a total of 1147 articles. Figure 1 provides a step-by-step illustration of why the titles were removed from the review process. Initially, the duplicate titles were removed (1147 to 878 articles). Then, the titles that did not include the target population or outcomes were removed (878 to 137 articles). Abstracts were then reviewed (*n* = 137) and those that did not meet the criteria were excluded (*n* = 93), leaving 44 full articles to be reviewed. After a full article review, the following articles were removed for not meeting criteria—(1) studied population outside the age range (*n* = 3), did not measure self-regulation (*n* = 17), did not measure or quantify PA (*n* = 6), was not an intervention (*n* = 4), or did not measure the children directly (used proxy report; *n* = 5), only methodology was published (*n* = 2), and unpublished data (*n* = 1). There were a total of six articles that were included in this review (See Figure 1). Studies included in this section examined at least one aspect of self-regulation in relation to PA, through an intervention in a preschool-aged population. These studies included a variety of measurements and focus within the aforementioned parameters. The studies are presented in the following order—(1) attention, (2) working memory, and (3) inhibition.

### 3.1. Attention

The following studies included the attention component of self-regulation in a preschool-aged population. Jarraya et al. examined the impact of kindergarten-based yoga on cognitive performance [19]. Subjects included 45 healthy children (5.2 ± 0.4 y, 17 males), who were randomly assigned to one of three groups—yoga (*n* = 15), a generic physical education program (*n* = 15), or a control group (*n* = 15). All participants in the yoga and physical education groups attend 2, 30-min sessions per week for 12 weeks. Participants completed subtests of the Developmental Neuropsychological Assessment (NEPSY) and the 2nd edition of NEPSY (NEPSY-2) protocols (two cognitive aspects of attention—speed and precision of detecting targets among distractors), both before and immediately after the 12-week programs. PA was documented by time spent in PA during the intervention. Regarding the speed of detection, yoga did not have a significant impact compared to the physical education program, but did have a significant positive impact compared to the control group. For the precision of detection, yoga had a significant positive impact compared to the physical education program as well as when compared to the control group. After corrections, the only significant interaction effect that remained was that yoga had an impact on the precision of detection in comparison to the control group (adj. *p* = 0.005; ηp^2^ = 0.164) [19]. The effect size was considered large and these findings demonstrated a positive impact of yoga on attention control. The main findings for this study were significant, i.e., there were improvements observed pre- to post-intervention in attention in the yoga group with no improvements observed in the physical education or control groups. The overall risk of bias was categorized as low, indicating a high quality of the study.

Another study conducted by Zach et al. examined whether PA improves inhibition (impulsivity) and attention in 123 preschool-aged children (5.1 ± 0.7 y, 60 males) [20]. These subjects were split among three groups—an experimental group with intervention-orienteering (*n* = 44), an experimental group with intervention-dance (*n* = 40), and a control group (*n* = 39). An assessment of attention was completed using the MOXO Continuous Performance Test (MOXO-CPT) at the pre- and post-intervention time-points. PA was described by amount of time (minutes per session, sessions per week) spent in PA throughout the duration of the intervention. Specifically, with regards to their findings on the component of attention, there were differences between the measurements time-points in the two groups (dance and control), as per the MOXO-CPT (*p* < 0.05; η^2^ = 0.112). Additionally, there was an interaction between attention and impulsivity (*p* < 0.05; η^2^ = 0.126), when the stimuli were accompanied by disturbances. The two experimental groups significantly improved from the pre- to post-intervention measurement, while no such improvement was demonstrated for the control group [20]. Overall, Zach et al. observed a positive impact of PA on attention in preschool-aged children. However, the effect sizes for these findings were small. The main findings pertaining specifically to attention for this study were that there were significant improvements observed pre- to post-intervention in attention, whereas no improvement was observed in the control group. The overall risk of bias was categorized as high, indicating that there might be issues with the quality of the study.

Burkart et al. examined the effects of a PA intervention on classroom behaviors in 71 preschool-aged children (3.8 ± 0.7 y) from low socioeconomic environments [21]. The children were randomized to a locomotor-based PA group (LB-PA) or unstructured free playtime group (UF-PA). These interventions were integrated and facilitated by the teachers (trained in how to properly execute the programs) who implemented these sessions for 30 min per day, 5 days per week, for 6 months. Behavioral measurements specific to attention were collected at baseline, 3 months, and 6 months, using the Behavior Assessment System for Children, 2nd edition (BASC-2). The children’s PA was also measured using the ActiGraph GT1M accelerometer placed on the participants’ waist. On average, attention problems scores increased over time in the UF-PA group, with significant decreases in inattention in the LB-PA group (3.91 points, *p* < 0.001; 1.59 points, *p* < 0.001, respectively) [21]. There was a weak, negative correlation between attention and inhibition (r = −0.31; *p* = 0.04), indicating a small effect size. Overall, Burkart et al. observed a positive impact of PA on attention and the risk of bias in this study was classified as low.

The following study was the only study in our review that explored the relationships among all cognitive aspects of self-regulation and PA. However, only the results pertaining to attention are presented in this section. The main findings pertaining to attention in this study were the improvements observed in the intervention group. The overall risk of bias was categorized as low.

Healey and Halperin included 25 children (3.9 ± 0.6 y, 19 males) and their parents in their Enhancing Neurobehavioral Gains with the Aid of Games and Exercise (ENGAGE) intervention [22]. The ENGAGE program involved parents and children playing a wide range of games targeting self-regulation on a daily basis, over a 5-week period. Behavioral assessments used to investigate the attention component of self-regulation were collected via the BASC-2. PA was documented based on time spent in PA outlined by the intervention. Although the study did not have a typical control group to determine whether changes were larger than those expected during normal development. They examined the data in comparison to longitudinal data in a similar population, to compare differences. The effect size of these findings was classified as medium (ηp^2^ = 0.248). The main findings of this study, pertaining specifically to attention for this study was that the imposed intervention might lead to improved attention. The overall risk of bias was categorized as unclear.

In all studies reviewed that included an assessment of attention (*n* = 4), there was a positive relationship with PA. This relationship was present regardless of the method of assessment of PA or attention and the quality of the study. Three of the four studies were considered high quality, as they were classified to have a low risk of bias [19,21,22]. One study had a high risk of bias, so the results of said study should be interpreted with caution [20].

### 3.2. Working Memory

The following studies examined the impact of PA on the working memory component of self-regulation. Wen et al. randomly assigned 57 preschool-aged children (4.4 ± 0.3 y, 31 males) to either an intervention group (*n* = 29), or a control group (*n* = 28) [23]. The protocol included three phases—pretest, a 10-week intervention, and posttest. During the 10 weeks, the children in the intervention and control groups shared the same classes and care service in the preschool, but children in the intervention group participated in an additional 20 min of a trampoline training program. Working memory was measured using the working memory span (WMS) task, cognitive flexibility through the flexible item selection (FIS) task, and PA was measured using ActiGraph GTM accelerometers. Results indicated that there were no significant differences in the WMS and FIS tests between the two groups tested post intervention (ηp^2^ = 0.01; small effect) [23]. The main finding of this study that specifically pertained to working memory was that there was no observed impact on working memory. The overall risk of bias was categorized as low. 

Healey and Halperin used the Stanford Binet-5 (SB-5) and NEPSY / NEPSY-2 to assess changes in working memory, following a PA intervention [22]. They found significant improvements in working memory, specifically there were significant main effects of time on working memory, indicating improvements in behavior (d = 0.768; medium effect size). The main findings of this study pertaining specifically to working memory was that the imposed intervention might lead to improved working memory. The overall risk of bias was categorized as unclear, indicating that there might be a risk of bias impacting the findings [22].

Only two studies included an assessment of working memory and the findings were mixed. Based on this limited number of studies, there was no clear association between working memory and PA.

### 3.3. Inhibition

The following studies examined the influence of PA on the inhibition component of self-regulation. Robinson, Palmer, and Bub conducted a study that included 113 preschool-aged children (4.3 ± 0.5 y, 56 males) enrolled in a federally funded preschool program, who were randomized into either a group that received a motor skill intervention (*n* = 68) or a control group (*n* = 45) [24]. Self-regulation was assessed using the delay of gratification snack task of the Preschool Self-Regulation Assessment. This task tested the children’s impulse control, which fit into the inhibitory control component of self-regulation. PA was documented by time spent in PA during the intervention. At the pre-test assessment, the children in both groups scored relatively high, indicating a good self-regulation (inhibitory control), but the intervention group maintained their ability to control their impulse when presented with a snack. The control group saw a decline in their scores during the post-intervention assessment. The effect size (d = 0.727) of this study was considered to be ‘medium’. The main findings of this study pertaining specifically to inhibition were that significant treatment effects were observed for inhibition in the participants assigned to the treatment group. The overall risk of bias was categorized as ‘low.’

Zach et al. examined whether PA improves inhibition (impulsivity) in preschool-aged children (5.1 ± 0.7 y, 60 males), and in the same study, they also referenced previously examining the role of attention [20]. The subjects were split among three groups—an experimental group with intervention-orienteering (*n* = 44), an experimental group with intervention-dance (*n* = 40), and a control group (*n* = 39). Assessments related to the measure of inhibition were completed using the MOXO-CPT at the pre- and post-intervention time-points. PA was documented by time spent in PA throughout the intervention time-period. There were differences in inhibition (impulsivity) (*p* < 0.05; η^2^ = 0.064), between the two measurement time-points in the two groups (dance and control), as per the MOXO-CPT, but the effect size was classified as small. Additionally, as previously stated, there was an interaction between attention and impulsivity (*p* < 0.05; η^2^ = 0.126) when the stimuli were accompanied by disturbances. The experimental groups significantly improved from the pre- to post-intervention measurement, while no such improvement was demonstrated for the control group [20]. The main findings of this study pertaining specifically to inhibition were the significant improvements observed pre- to post-intervention versus no improvement in the control group. As previously mentioned, the overall risk of bias was categorized as high.

Burkart et al. examined the effects of a PA intervention on classroom behaviors in 71 preschool-aged children (3.8 ± 0.7 y) from low socioeconomic environments [21]. The children were randomized to a locomotor-based PA group (LB-PA) or unstructured free playtime group (UF-PA). These interventions were integrated and facilitated by the teachers (trained in how to properly execute the programs) who implemented these sessions for 30 min per day, 5 days per week, for 6 months. Behavioral measurements were collected at baseline, 3 months, and 6 months, using the BASC-2 (attention) and a computerized Go / No-Go task (inhibition). The children’s PA was measured using the ActiGraph GT1M accelerometer placed on the waist and positioned on the lower back. On average, attention problem scores increased over time in the UF-PA group, with significant decreases in inattention in the LB-PA group (3.91 points, *p* < 0.001; 1.59 points, *p* < 0.001, respectively) [21]. The main finding of this study, pertaining specifically to inhibition, was that there was no observed impact of the intervention on inhibition. The overall risk of bias was categorized as low.

Wen et al. also examined the role of working memory on PA, while also examining the role of inhibition [23]. Inhibitory control was measured using the spatial conflict arrow (SCA) task and the Animal Go/NoGo (GNG), and PA was measured using the ActiGraph GTM accelerometers. Their results indicated that there were no significant differences in the SCA (ηp^2^ = 0.04) or GNG (ηp^2^ = 0.03) tests between the two groups tested post intervention. The main finding of this study, pertaining specifically to inhibition, was that the results indicated that no significant improvements were observed in inhibition, following the imposed intervention. The overall risk of bias was categorized as low.

Lastly, Healey and Halperin found significant improvements in inhibition as a result of the PA intervention [22]. Inhibition was assessed using the NEPSY/NEPSY-2 [22]. PA was documented based on time spent in PA outlined by the intervention. When examining whether children’s time spent playing the intervention games was associated with changes in behavior, only the correlation with inhibition was significant. Significant main effects of time on all three measures of self-regulation indicated improvements in cognitive behaviors. The ENGAGE group performed poorly on the measures of inhibition at pre-test, but that performance improved significantly over time (d = 0.708; medium effect) [22]. The main findings of this study pertaining specifically to inhibition was that the imposed intervention might lead to improved inhibition. The overall risk of bias was categorized as unclear.

The result from these studies appear to be inconclusive with three out of five studies finding a positive association between inhibition and PA. The lack of consensus might be due to the variety of assessment modes of inhibition and PA.

## 4. Discussion

The purpose of this review was to examine the relationships between self-regulation, including the separate cognitive aspects of attention, working memory, inhibition, and PA, in preschool-aged youth. Only one study, incorporated all cognitive aspects of self-regulation and found that there were improvements in attention, working memory, and inhibition, as result of the PA intervention [22]. In the current review, attention was the only component of self-regulation that consistently showed improvement as a result of the PA interventions [19,20,21,22]. The findings for working memory and inhibition were inconclusive regarding changes in these variables with PA. Half of the studies (1 of 2) showed a positive impact of PA on working memory, and three of five studies found a positive impact of PA on inhibition [20,22,24].

### 4.1. Attention

All studies in this review that included an assessment of attention (*n* = 4), found a positive association with PA. In young children, specifically around two years of age, attention was the first of the cognitive aspects of self-regulation to develop [25]. Then closer to the age of 3 years, children developed the ability to exhibit control over their behaviors. The age of the children in these studies ranged from 3–6 years old. During this timeframe, attention development had begun and was shown to increase with age, indicating that this population had sufficient attention development to establish a relationship with PA [25].

In the current literature, the relationship between attention and PA is well-established in older youth [26,27]. It appears that the impact that PA has on attention begins in early childhood and continues to have a positive effect throughout childhood and adolescence. Attention is key to the continued development of self-regulation and cognitive function. Structured activity, such as games and sports, were shown to influence activity in the prefrontal cortex [15]. The prefrontal cortex is the area of the brain that is associated with attention and the ability to hold sustained attention. All interventions that included a measure of attention consisted structured activities, such as games, orienteering, and specific motor activities. It is possible that the consistent findings of improved attention with PA could be due to the fact that the activities in the interventions stimulated the prefrontal cortex, resulting in an improved ability to sustain attention during the cognitive tasks.

### 4.2. Working Memory

Working memory develops subsequent to attention in young children, usually within the first three years. Working memory is crucial to learning and utilizing previously acquired information, to execute more complex cognition functions, such as problem solving and comprehending information presented [28,29]. In the current review, working memory was the least studied component of self-regulation (*n* = 2), and the findings were inconclusive. The Wen et al. study found no change in working memory as a result of a mini-trampoline intervention [23]. This finding suggests that this type of activity, similar actions repeated over time, jumping on a trampoline, might not be complex enough to stimulate the development of neural connections that are necessary to elicit changes in cognitive function, specifically working memory.

Healey and Halperin conducted an intervention composed of activities and games that integrated the targeted cognitive skills in the study [22]. Examples of the activities included: ‘Object Copy’—observe a structure being built and re-create the structure from memory and ‘Ball Games’—focus on a ball and catch it following various sequences. These activities not only targeted working memory but also included complex motor skills (i.e., catching a ball). The stimulation of the prefrontal cortex that was elicited during these activities might have contributed to the positive impact that this intervention had on working memory. These findings were consistent with the results from the studies in this review related to attention. All interventions that specifically focused on attention, included activities that were complex in nature and involved a variety of movement patterns. These findings indicate that specific types of activity might be required, in order to see the impact of PA on working memory. Additionally, the method of assessment of working memory could have influenced the results of the studies. The WMS was used in the Wen et al. study and was adapted for children from a tool designed for adults [23]. The SB-5 assessment was used in the Healey and Halperin study, and was designed specifically for children, which might increase the sensitivity of the test and could lead to significant findings [22]. It appears the type of activity as well as the assessment of working memory might influence findings. Due to the limited number of studies and mixed results from those studies, it is too difficult to discern a clear relationship or explanation of the results. This area needs further exploration in order to determine whether a relationship exists between PA and working memory in young children.

### 4.3. Inhibition

Inhibition was assessed in the majority of the studies included in this review (5 of 6). Inhibition is the ability to ignore irrelevant information while focusing on a relevant task. Inhibition is the most commonly assessed component of self-regulation and the assessment of inhibition was combined with assessments of other cognitive aspects of self-regulation, in all but two studies in this review. Inhibition is the most complex component of self-regulation and necessitates adequate working memory and attention skills. Due to the age of the participants in these studies, it is possible that the young children might not have had sufficient cognitive development to fully comprehend the tasks they were asked to complete. In early childhood, inhibitory control might be limited as other executive functions (such as attention and working memory) develop [30,31]. As attention and working memory develop in young children, inhibitory control follows. Children in the lower limit of the targeted age range (3-years old), typically have very poor inhibitory control; however, as the children age and the other cognitive aspects of self-regulation develop, so does inhibition [32].

Three of the five studies reviewed showed a relationship between inhibition and PA [20,22,24]. Each of these three studies included young children with a varied age range (3.8–5.07 years), included inhibition assessments developed for young children, and all but one included complex, varied activity as part of the intervention. It is possible that the relationship was significant in the Zach et al. and Robinson et al. study due to the age of the children [20,24]. The Zach et al. (5.07 years) and Robinson et al. (4.3 years) studies included older children, as well as activities that stimulated the prefrontal cortex, possibly improving self-regulatory skills [20,24]. Although the children in the Healey and Halperin were on the younger end of the range (3.9 years), the intervention included activities that specifically targeted self-regulation skills, as previously mentioned [22]. Specifically, the game ‘Simon Says’ was included, and the combination of PA and tasks that work on inhibitory control and might have influenced the relationship between PA and inhibition, despite the younger age of the children. The children in the Wen et al. study were near the top end of the age range (4.4 years), however; it is possible that a relationship was not found due to the type of activity utilized in the intervention (mini trampoline) [23]. Significance might not have been found in the Burkart et al. study due to the age of the children (3.8 years) [21]. Burkart et al. hypothesized that the classic Go/No-Go assessment might not have been appropriate for the targeted population in this study [21]. It appears that the intervention activities used in this study seem to be appropriate to stimulate improvement in cognitive function; however, a significant relationship existed between attention and PA but not inhibition and PA. It appears that age of the child, the assessment, and the type of activity might impact the relationship between inhibition and PA in young children, as young children might not yet have developed sufficient cognitive skills to complete the inhibition tasks and less complex PA tasks might not provide sufficient stimulation to cause improvements in inhibitory control.

### 4.4. Strengths and Limitations 

There are several strengths of this review. All studies incorporated a PA intervention in preschool-aged children to examine possible relationships with self-regulation and its cognitive aspects. Additionally, the majority of studies included in this review (66.67%) were classified as low risk of bias, one study was classified as unclear risk of bias, and only one study was classified as high risk of bias. Since the majority of the studies had a low risk of bias, they were deemed to be high quality. Thus, due to the high quality of the papers reviewed, the findings appear to represent the associations among PA and the cognitive aspects of self-regulation. This review aids to the sparse current body of research in this area, by highlighting the current methodological practices in assessment of self-regulation and its cognitive aspects, in comparison to PA in preschool-aged children. Although there was only a small number of studies included in this review, it brings to light the factors that need to be considered when looking at the impact of PA on self-regulation and in the design of studies and intervention programs. Limitations included the variability in the way PA was measured, the type of activities that were implemented in the interventions, and the manner of assessment of self-regulation and its cognitive aspects. Due to the lack of consistency, it is difficult to ascertain the ideal interventions that should be implemented in young children to improve self-regulation. The preschool-aged population is also a relatively understudied one with few publications that could be included, which examined the possible relationship between these variables. Additionally, a limitation to note is that this review was not registered in PROSPERO or any international database of prospectively registered systematic reviews, prior to the inception of the review process.

## 5. Conclusions

Overall, the findings from this review indicate that the only component of self-regulation that was consistently related to PA was attention. The existence of a clear relationship was not evident between working memory, inhibition, and PA. As a young child ages and develops, cognitive functions, such as attention, working memory, and inhibition, improve over time. This is evident in the middle childhood and early adolescent time period, as studies have found that physical activity and sports participation is related to improved cognitive development and self-regulatory skills [15,16]. These studies demonstrated that the relationships between physical activity, cognitive development, and self-regulation continues to develop, as a young child moves through childhood into adolescence. As the cognitive aspects of self-regulation build upon each other to develop the more complex executive functions, it might be necessary to assess each of these cognitive aspects in young children, as they develop at different stages of early childhood. The findings of this review suggest that assessment through multiple tools might provide a more in-depth picture of the children’s self-regulation, with the caveat of being a more time-consuming venture. Future research might use these results to determine the appropriate approach for assessing self-regulation, whether the purpose is an in-depth assessment of the cognitive aspects of self-regulation, or a broader snapshot of self-regulation as a whole. This review highlights the complexities and inconsistencies of this area of research in young children, supporting the need for further research in the area of self-regulation and all its cognitive aspects in this population.

## Figures and Tables

**Table 1:** The effects of physical activity on cognitive self-regulation in preschool-aged children.

Author/Year	Participant Information	Component of Self-Regulation	Assessment Tools	Physical Activity	Main Findings	Risk of Bias	Overall Impact
Burkart et al. (2018)	*n* = 71 (3.8 ± 0.7 y)	Inhibition and Attention	BASC-2, Go/No-Go	30 min per day, 5 days per week for 6 months, locomotor-based activity and unstructured free play groups ActiGraph GT1M accelerometer	No impact of intervention on inhibition. Attention was improved in the intervention group.	Low	Inhibition (0) Attention (+)
Healy & Halperin (2014)	25 families (3.9 ± 0.6 y)	Inhibition, Attention, and Working Memory	BASC-2, NEPSY-2, SB-5	Games and Exercises with family – 30 min daily – 5 weeks	Intervention might lead to improved attention, working memory, and inhibition.	Unclear	Inhibition (+) Attention (+) Working Memory (+)
Jarraya et al. (2019)	*n* = 45 (5.2 ± 0.4 y)	Attention	NEPSY, NEPSY-2	30 min per day, 2 days per week for 12 weeks of Yoga and 30 min per day, 2 days per week for 12 weeks of physical education	Significant improvements were observed pre- to post-intervention in attention in the Yoga group. No improvement was observed in the physical education or control group	Low	Attention (+)
Robinson et al. (2016)	*n* = 113 (4.3 ± 0.5 y)	Inhibition	Delay of gratification snack task of the Preschool Self-Regulation Assessment	15, 40-min sessions (3× per week for 5 weeks)	Significant treatment effects were found for self-regulation score (Inhibition) for the participants in the treatment group.	Low	Inhibition (+)
Wen et al. (2018)	*n* = 57 (4.4 ± 0.3 y)	Inhibition and Working Memory	SCA, GNG, WMS, FIS	Trampoline Intervention – 9 weeks, ActiGraph GT1M Accelerometer	Findings indicated that no significant improvements were found in the inhibitory control and working memory, following a 10-week trampoline PA training in preschool children.	Low	Inhibition (0) Working Memory (0)
Zach et al. (2015)	*n* = 123 (5.1 ± 0.7 y)	Inhibition and Attention	MOXO-CPT	Orienteering Group – 9 weekly activities – Dance – Ten dances – Control group – Recess	Significant improvements were observed pre- to post-intervention in attention, whereas no improvement was observed in the control group.	High	Inhibition (+) Attention (+)

MOXO-CPT: MOXO Continuous Performance Test; BASC-2: Behavior Assessment System for Children, 2nd edition; NEPSY: Developmental Neuropsychological Assessment; NEPSY-2: Developmental Neuropsychological Assessment, 2nd edition; SCA: Spatial conflict arrow; GNG: Animal Go/NoGo; WMS: Working memory span; FIS: Flexible item selection; SB-5: Stanford Binet; + = Positive Impact; – = Negative Impact; 0 = No Impact.

**Table 2:** Risk of Bias Assessment [18].

Author/Year	Risk of Bias							Overall Risk of Bias
Burkart et al. (2018)	Low	High	High	?	Low	Low	Low	Low
Healy & Halperin (2014)	?	?	?	?	Low	Low	High	?
Jarraya et al. (2019)	Low	Low	?	Low	Low	Low	?	Low
Robinson et al. (2016)	Low	?	?	Low	High	Low	Low	Low
Wen et al. (2018)	Low	Low	Low	Low	Low	Low	Low	Low
Zach et al. (2015)	High	High	High	?	?	Low	Low	High
	Random Sequence Generation	Allocation Concealment	Blinding of Participants and Personnel	Blinding Outcome Assessment	Incomplete Outcome Data	Selective Reporting	Other Bias	
								Legend
								Low = Low Risk
								High = High Risk
								? = Unclear Risk
